# Comprehensive Analysis of the Relationships Between the Gut Microbiota and Fecal Metabolome in Individuals With Primary Sjogren’s Syndrome by 16S rRNA Sequencing and LC–MS-Based Metabolomics

**DOI:** 10.3389/fimmu.2022.874021

**Published:** 2022-05-11

**Authors:** Li Yang, Zhao Xiang, Jinmei Zou, Yu Zhang, Yuanpiao Ni, Jing Yang

**Affiliations:** ^1^ Department of Rheumatology, Mianyang Central Hospital, School of Medicine, University of Electronic Science and Technology of China, Mianyang, China; ^2^ Department of Clinical Medicine, North Sichuan Medical College, Nanchong, China

**Keywords:** primary Sjogren’s Syndrome, gut microbiota, 16S rRNA sequencing, metabolome, LC–MS, *Escherichia-Shigella*

## Abstract

The gut microbiota has been associated with primary Sjogren’s syndrome (pSS), yet the biological implications of these associations are often elusive. We analyzed the fecal microbiota through 16S rRNA gene amplification and sequencing in 30 patients with pSS and 20 healthy controls (HCs); At the same time, the fecal metabolome was characterized by ultrahigh-performance liquid chromatography–mass spectrometry. In addition, correlation analyses of microbiota and metabolome data were performed to identify meaningful associations. We found that the microbiota composition of pSS patients was significantly different from that of HCs. The pSS gut microbiota is characterized by increased abundances of proinflammatory microbes, especially *Escherichia-Shigella*, and decreased abundances of anti-inflammatory microbes. Concerning the metabolome, a multivariate model with 33 metabolites efficiently distinguished cases from controls. Through KEGG enrichment analysis, we found that these metabolites were mainly involved in amino acid metabolism and lipid metabolism. The correlation analysis indicated that there were certain correlations between the microbiota and metabolism in pSS patients. In addition, an abundance of *Escherichia-Shigella* was found to be correlated with high levels of four metabolites (aflatoxin M1, glycocholic acid, L-histidine and phenylglyoxylic acid). Our research suggests that in pSS patients, the gut microbiota is characterized by a specific combination of proinflammatory changes and metabolic states. *Escherichia-Shigella* is a factor related to gut dysbiosis, which may promote intestinal damage and affect amino acid metabolism.

## 1 Introduction

Primary Sjogren syndrome (pSS) is one of the most common autoimmune diseases and is characterized by focal lymphocytic infiltration of the exocrine glands, which can result in glandular dysfunction and sicca symptoms.Additionally, 67% of pSS patients were found to have fatigue (1) and 36.9% of pSS patients had depression (2). These symptoms can significantly decrease quality of life. In addition, the disease can also lead to various extraglandular effects, including effects on the articular, pulmonary, renal and nervous systems (3). Once important organs are involved, the disease poses a threat to life.

Recent studies show that the occurrence of pSS is closely related to genetic susceptibility (4, 5), environmental factors (6), immune abnormalities (7) and other factors.

Recently, an increasing number of studies have focused on the gut microbiota. The gut microbiota plays an important role in maintaining intestinal homeostasis. It can induce the development of the intestinal immune system through methods, including the promotion of intestine-associated lymphoid tissue development and the production of DCs, Th17 cells, regulatory T cells, plasma cells and innate lymphocytes (8, 9). In addition, short-chain fatty acids (SCFAs), which are fatty sugars secreted by the gut microbiota, can protect the intestinal mucosal barrier and regulate intestinal immunity.One study on the gut microbiota associated with pSS showed that *Firmicutes* was the dominant phylum in the gut, and at the phylum level, pSS patients exhibited depletion of *Firmicutes* and enrichment of *Proteobacteria*, *Actinobacteria*, and *Bacteroidetes* compared to the controls, which indicated that there were gut microbiota alterations associated with pSS (1). Moon et al.’s (10) research showed that SS patients had significant gut dysbiosis compared to controls, and there was a certain correlation between the gut dysbiosis and the severity of dry eye. Another study showed that compared with controls, pSS patients owned more serious gut microbiota dysbiosis. This study showed subjects with pSS and severe dysbiosis had higher disease activity, lower complement 4 levels and higher fecal calprotectin levels than the other pSS patients, indicating that severe intestinal dysbiosis is prevalent in pSS patients and it is associated with systemic disease activity and gastrointestinal inflammation (11).

In addition, two studies focused on the dysbiosis of the buccal mucosal microbiota in pSS and found that pSS patients had a relatively higher *Firmicutes*/*Proteobacteria* ratio and a lower relative abundance of *Streptococcus* (12, 13).

It is being increasingly recognized that the gut microbiota and its products may affect the immune response. One study on the blood metabolites and the intestinal microbiota of rheumatoid arthritis (RA) patients showed that the overall metabolites differed significantly between RA patients and controls, and the metabolites were correlated significantly with the microbiota of RA patients (14). Another study conducted on systemic autoimmune diseases (SADs), including SLE, SS, and primary anti-phospholipid syndrome (PAPS), indicated that in all of the SADs, the abundance of protolerogenic bacteria was reduced, while the abundance of pathobiont genera was increased. Metabolic analysis showed that SADs patients owned particular metabolomic characteristics that could distinguish them from healthy controls (HCs). This research indicated that there was a strong interaction between the gut microbiota and metabolic function in SAD patients (15). The metabolomics study performed by Li et al. (16) in SS patients in comparison with RA patients showed that no definite conclusion could be made.

To date, very few studies have explored the gut microbiota and metabolites associated with SS. In our study, we analyzed the gut microbiota and fecal metabolites in patients with SS. We hypothesize that pSS patients may have different microbiota features and metabolites.

## 2 Material and Methods

### 2.1 Patients and Controls

Thirty pSS patients in the Department of Rheumatology, Mianyang Central Hospital, Sichuan, China and fulfilling the American-European Consensus Group (AECG) criteria (17) and the American Congress of Rheumatology-EULAR criteria for pSS (18) were included. All patients were newly diagnosed and untreated. Twenty age- and sex-matched healthy controls were included. The P-value of the analysis of the difference in age across the group is 0.1, which showed no statistical difference. Exclusion criteria included concurrent IBD/severe diarrhea and antibiotic treatment during the last 3 months. The subjects’ characteristics are presented in **Table 1**.

**Table 1:** Demographic and clinical characteristics of the study participants.

	pSS (n = 30)	HCs (n = 20)
Age, mean (SD)	55.5 (8.80)	50.7 (10.1)
Female, n (%)	29 (96.7)	19 (95.0)
Disease duration, years, median (interquartile range)	2.03 (6.84)	-
ESSDAI, median (interquartile range)	4.00 (5.00)	-
Involved system, n (%)		-
Lymphadenopathy	1 (3.33)	
Domain	2 (6.67)	
Glandular domain	5 (16.7)	
Articular domain	2 (6.67)	
Cutaneous domain	4 (13.33)	
Pulmonary domain	1 (3.33)	
Renal domain	0 (0)	
Muscular domain	0 (0)	
Peripheral nervous system domain	1 (3.33)	
Central nervous system domain	10 (30.33)	
Hematological domain	23 (76.67)	
Biological domain		

ESSDAI, EULAR Sjogren’s syndrome disease activity index.

### 2.2 Clinical Assessment and Laboratory Analyses

Disease activity of the pSS patients were evaluated by the ESSDAI (19).

The levels of autoantibodies, including the anti-SS-A, anti-Ro52, and anti-SS-B antibodies and ANA, were measured and recorded. All analyses were performed at the Department of Laboratory Medicine, Mianyang Central Hospital.

### 2.3 Sample Collection and Storage

The plasma samples from patients were frozen at -20°C until delivery to the Department of Laboratory Medicine for detection. Fecal samples from pSS patients and HCs were frozen at -80°C within 4 hours of receipt until delivery to the laboratory for processing and analysis.

### 2.4 Fecal Microbiota Analysis

Fecal microbiota analysis was performed by PCR amplification.

#### 2.4.1 Extraction of Genomic DNA

Total genomic DNA was extracted by the CTAB method. DNA concentration and purity were monitored on 1% agarose gels. Sterile water was used to dilute the DNA to 1ng/L based on the original DNA concentration.

#### 2.4.2 Amplification Generation

Specific primers(515F-806R) were used to amplify the 16S rRNA genes of distinct regions (16S V4/16S V3/16S V-V4/16S V4-V5). All PCRs assays were performed with 15 µL of Phusion ^®^ High-Fidelity PCR Master Mix (New England Biolabs). For thermal cycling, initial denaturation was performed at 98°C for 1 minute, followed by denaturation at 98°C for 10 seconds, annealing at 50°C for 30 seconds, and extension at 72°C for 30 seconds and circulation for 30 times. Finally, the samples were incubated at 72°C for 5 minutes.

#### 2.4.3 PCR Product Quantification and Qualification

An equal volume of 1X loading buffer (including SYBR green) was mixed with PCR products, and electrophoresis was performed on a 2% agarose gel for detection. PCR products were mixed at isodensity ratios. The PCR products were then purified using the Qiagen Gel Extraction Kit (Qiagen, Germany).

#### 2.4.4 Library Preparation and Sequencing

TruSeq^®^ DNA PCR-Free Sample Preparation Kit (Illumina, USA) was used for library construction. The constructed library was quantified by Qubit@ 2.0 Fluorometer (Thermo Scientific) and an Agilent Bioanalyzer 2100 system. After the library was qualitatively assessed, Illumina NovaSeq6000 was used for machine sequencing.

### 2.5 Fecal Metabolomic Analysis

One hundred mg of each fecal samples was placed into an EP tube and 500uL of 80% methanol aqueous solution was added. The samples were incubated on ice for 5 minutes and then centrifuged at 4°C at 15,000 ×g for 20 minutes. A certain amount of supernatant was diluted with LC-MS grade water until the methanol content was 53%. Centrifugation was performed at 15000 ×g and 4°C for 20 minutes. Finally, the supernatant was collected and injected into the LC–MS system for analysis. Equal volumes were taken from each sample and mixed as quality control (QC) samples.

UHPLC–MS spectrometry was performed using a Vanquish UHPLC system (Thermo Fisher, Germany) combined with an Orbitrap Q ExactiveTM HF-X mass spectrometer (Thermo Fisher, Germany). Samples were injected onto a Hypersil Gold column (100×2.1 mm, 1.9 μm) at a flow rate of 0.2 mL/min with a linear gradient of 17-min. The positive polarity eluents were eluent A (0.1% formic acid (FA) in water) and eluent B (methanol). The negative polarity eluents were eluent A (5 mM ammonium acetate, pH 9.0) and eluent B (methanol). The solvent gradient was set as follows: 2% B, 1.5 minutes; 2-100% B, 12.0 minutes; 100% B, 14.0 minutes; 100-2% B, 14.1 minutes; 2% B, 17 minutes.

### 2.6 Data Analysis

#### 2.6.1 Analysis of the Microbiota

Clustering of the sequences and grouping according to operational taxonomic units (OTUs) were performed. UPARSE software (UPARSE v7.0.1001) was used to cluster all effective tags of all samples and the sequence was clustered into the same OTUs with 97% consistency. Through comparison with the Silva138 database, species annotation and statistics of different classification levels were carried out. Alpha diversity was used to analyze the community richness and community diversity through Chao1 and the Shannon index. QIIME (Version 1.7.0) was used to calculate UniFrac distance and to construct a UPGMA sample clustering tree. R software (Version 2.15.3) was used to draw PCA and PCoA diagrams. Beta diversity analysis, which was performed based on weighted UniFrac distances and calculated by QIIME software (Version 1.9.1) was used to evaluate differences in species complexity. Before cluster analysis, principal component analysis (PCA) was performed, and variables that were originally dimensionless were reduced using the FactoMineR package and ggplot2 package in R software (Version 2.15.3). Principal coordinates were obtained by principal coordinate analysis (PcoA) to visualize complex multidimensional data. Linear discriminant analysis (LDA) effect size (LEfSe) is an analytical tool to discover and interpret high-dimensional biomarkers. It particularly emphasizes statistical significance and biological correlation and is able to identify biomarkers that differ significantly between groups. It was performed using the LEfSe tool available in the public domain. Only when the p value was < 0.05 and the log LDA score was ≥ 4, were those taxa ultimately considered. Random forest, proposed by Breiman (20), is a classic machine learning model based on the classification tree algorithm, which has important applications in pattern recognition. By dividing the data into training sets and test sets and training the classification function continuously, the optimal classification effect could be achieved. Then, the classification effect was verified with the test set. In ecological studies, random forest algorithms are mainly used to build classification models and screen biomarkers that play an important role in classification or grouping.

#### 2.6.2 Metabolomic Analysis

The raw data files generated by UHPLC–MS/MS were imported into Compound Discoverer 3.1 (CD3.1, Thermo Fisher) for processing. The retention time, mass charge ratio and other parameters of each metabolite were simply screened. Then the retention time deviation of 0.2minutes and quality deviation of 5PPM were set for peak alignment of different samples to make identification more accurate. Then, peak intensities were normalized to the total spectral intensity. The normalized data were applied to molecular formula prediction based on additive ions, molecular ion peaks and fragment ions. Then, peak matching was performed with the mzCloud, mzVault and MassList databases, in order to obtain accurate qualitative and relatively quantitative results. Data processing was performed in R software (R version R-3.4.3), Python (Python 2.7.6 version) and CentOS (CentOS release 6.6). When the data were not normally distributed, the area normalization method was tried for normal transformation.

The KEGG database, HMDB and LIPIDMaps database were used to annotate the identified metabolites. For multivariate statistical analysis, MetaX was used to transform the data and then perform the principal component analysis (PCA) and partial least squares discriminant analysis (PLS-DA). For univariate analysis, the statistical significance (P value) of each metabolite between the two groups was calculated based on the T test. The fold change (FC value) of the levels of metabolites between the two groups was calculated. Metabolites with VIP > 1, P value< 0.05 and fold change (FC)≥2 or FC ≤ 0.5 were considered to be differential metabolites.

The volcano map was drawn with R package (ggplot2), which could integrate the VIP value, log2 (FoldChange) and log10(p value) to screen the metabolites of interest. Clustering heatmaps were drawn by Pheatmap package in R language, and metabolites data were normalized by z scores. Cor.mtest() in R language was used to calculate statistically significant correlations between differential metabolites. Cor() in R language (method=Pearson) was applied to analyse the correlation between differential metabolites. Corrplot package of R language was used to draw the correlation plots. A P value of < 0.05 was considered statistically significant. The KEGG database was used to study the functions and metabolic pathways of these metabolites. Moreover, metabolic pathway enrichment of differential metabolites was performed. The metabolic pathway was considered enriched when x/n > y/N was satisfied. Additionally, when the P value of a metabolic pathway was < 0.05, the metabolic pathway was considered to be significantly enriched.

## 3 Results

### 3.1 Clinical and Demographic Characteristics of the Study Participants

Most of the pSS patients were female. The mean disease activity assessed by ESSDAI was 4.6. The mean disease duration was 3.69 years. The clinical and demographic characteristics of the study participants were shown in **Table 1**.

### 3.2 Gut Microbiota Analysis

#### 3.2.1 Alpha Diversity and Beta Diversity of the Gut Microbiota

Alpha diversity represents the species evenness and richness within the microbiota, while beta diversity represents the shared diversity within the microbiota at different ecological distances (21).

**Figure 1:**
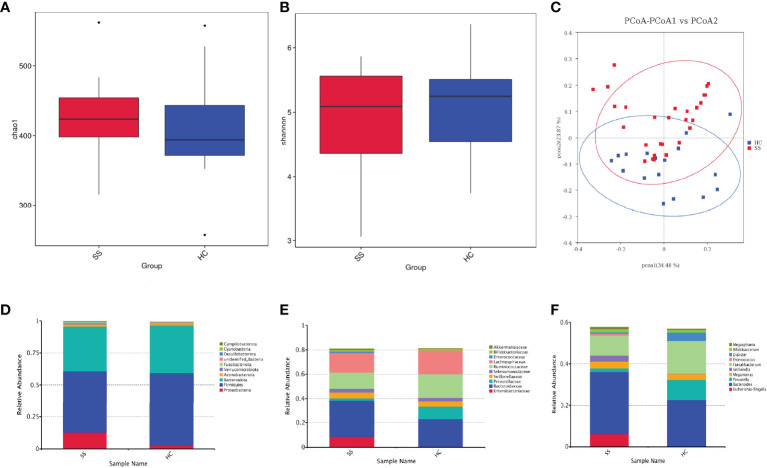
The gut microbiota of pSS patients differs from that of HCs and taxonomic proportions according to the compositions of the phyla, families and genera of pSS patients and HCs. **(A)** Chao 1 index of pSS patients and HCs. **(B)** Shannon diversity index of pSS patients and HCs. **(C)** Beta diversity of the genera analyzed by weighted UniFrac PCoA. **(D)** Taxonomic proportions according to compositions at the phylum level. **(E)** Taxonomic proportions according to compositions at the family levels. **(F)** Taxonomic proportions according to compositions at the genus levels.

First, we analyzed the alpha diversity of the gut microbiota of the two groups. The Chao 1 index describing species richness revealed no significant differences (pSS vs. controls; p=0.360, **Figure 1A**). In addition, the Shannon diversity index did not show significant differences between the two groups (pSS vs. controls; p=0.394, **Figure 1B**).

Then, we evaluated the beta diversity of the two groups. Through weighted UniFrac PCoA, we observed some differences in species classification between pSS patients and HCs (**Figure 1C**), but there was no statistical difference

Through alpha and beta diversity analysis, we showed the gut microbiome was similar between tbe healthy controls and patients with pSS.

#### 3.2.2 Alteration in the Microbiota

There were a total of 520108716S rRNAs identified from the analysis and 1609 OUTs were found, among which the number of OTUs that could be annotated from the database was 1,607 (99.88%), with 99.88% at the kingdom level, 93.54% at the phylum level, 92.73% at the class level, 89.81% at the order level, 84.21% at the family level, 63.21% at the genus level, and 19.89% at the species level. We examined the compositional differences in the gut microbiota at the phylum, family and genus levels between the two groups (**Figures 1D–F**). pSS patients showed marked differences in composition compared to the HCs.

At the phylum level, *Firmicutes* was the dominant member of the gut microbiota in all individuals, constituting between 40% and 60% of all phyla, followed by *Bacteroidetes* (30%-40%). pSS patients exhibited depletion of *Firmicutes* (1.2-fold) and *Bacteroidota* (1.1-fold) and enrichment of *Proteobacteria* (4.4-fold), *Actinobacteriota* (1.3-fold), *Verrucomicrobiota* (7.4-fold), and *Fusobacteriota* (2.7-fold) relative to the levels in the HC. pSS patients had a lower *Firmicutes*-*Bacteroidetes* ratio than the controls (1.37/1.52).

The families *Enterobacteriaceae* (15.1-fold) and *Bacteroidaceae* (1.35-fold) were enriched in pSS patients; in contrast, *Ruminococcaceae* (1.5-fold) and *Lachnospiraceae* (1.2-fold) were depleted.

At the genus level, pSS patients showed significantly increased abundances of *Escherichia-Shigella* (19.5-fold), *Veillonella* (15.4-fold) and *Bacteroides* (1.3-fold). Additionally, pSS patients exhibited reduced abundances of the genera *Faecalibacterium* (1.7-fold) and *Prevotella* (5.7-fold) compared to the control group.

#### 3.2.3 Diagnostic Biomarkers for pSS in the Gut Microbiota

The statistical results of LEfSe included three parts, namely, a histogram of the LDA value distribution, a cladogram, and a comparison of the abundances of significantly different biomarkers in different groups.

**Figure 2:**
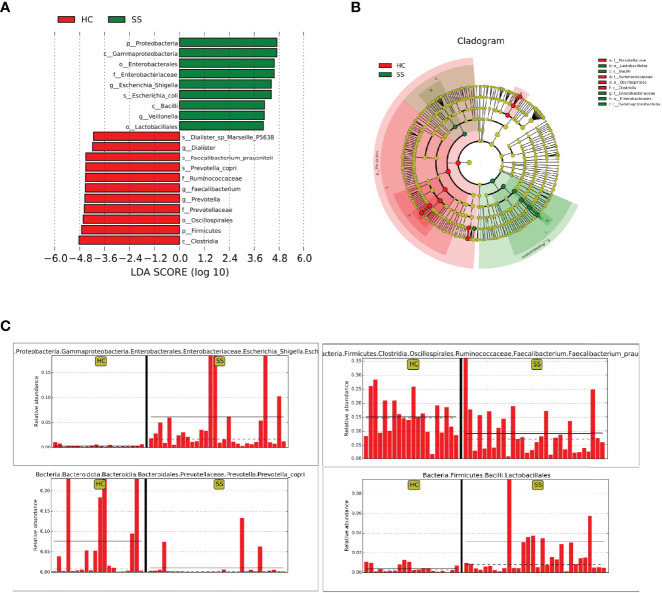
LEfSe analysis. **(A)** Histogram of LDA value distribution. **(B)** Cladogram. **(C)** Comparison of the abundances of significantly different biomarkers (*Escherichia-Shigella, Lactobacillales, Faecalibacterium, Prevotella*) in pSS patients and HCs.

The histogram of the LDA value distribution (**Figure 2A**) showed that there were 20 differentially abundant taxa at different taxonomic levels, of which 9 were from pSS patients and 11 were from HCs. The class *Clostridia* had the largest LDA score.

The cladogram (**Figure 2B**) showed that the abundance of *Enterobacteriaceae* from the phylum *Proteobacteria* was increased in patients compared with HCs. In addition, *Lactobacillales* from *Bacilli* also clustered differently and was enriched in pSS patients. The relative abundances of some significantly different biomarkers are shown in **Figure 2C**. *Escherichia-Shigella* from *Enterobacteriaceae* and *Lactobacillales* from *Bacilli* were significantly enriched in pSS patients compared with controls. In contrast, *Faecalibacterium prausnitzii* from *Clostridia* and *Prevotellaceae* from *Bacteroidota* were enriched in controls compared with pSS patients.

**Figure 3:**
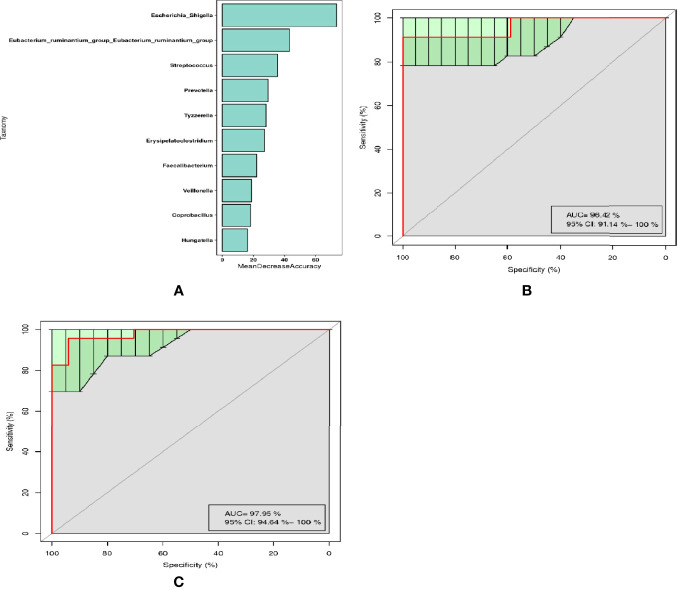
Diagnostic biomarker analysis by a random forest model: **(A)** Random forest model of the ten microbial genera model. **(B)** ROC curve of the ten microbial model. **(C)** ROC curve of the three microbial genera model.

Through random forest analysis, a simplified model comprising ten microbial genera (**Figure 3A**) was able to discriminate pSS patients from HCs with an overall AUROC of 96.42% (95%CI: 91.14%-100%) (**Figure 3B**), of which the genera *Escherichia-Shigella*, *Eubacterium-ruminantium, and Streptococcus* were confirmed to have significantly more discriminatory power than that of others, with an overall AUROC of 97.95% (95% CI: 94.64%-100%) (**Figure 3C**), indicating that they were good diagnostic markers. In addition, *Escherichia-Shigella* from the family *Enterobacteriaceae* showed the highest diagnostic value, which was in accordance with the LEfSe results.

### 3.3 Fecal Metabolic Differences Between pSS Patients and HCs

In the present study, a non-targeted metabolomics study was carried out based on LC-MS technology. The experimental procedures mainly included sample preparation, metabolite extraction, and LC/MS detection and data analysis. A total of 50 serum samples and 6 QC samples were analyzed by UHPLC-Q-TOF/MS.

**Figure 4:**
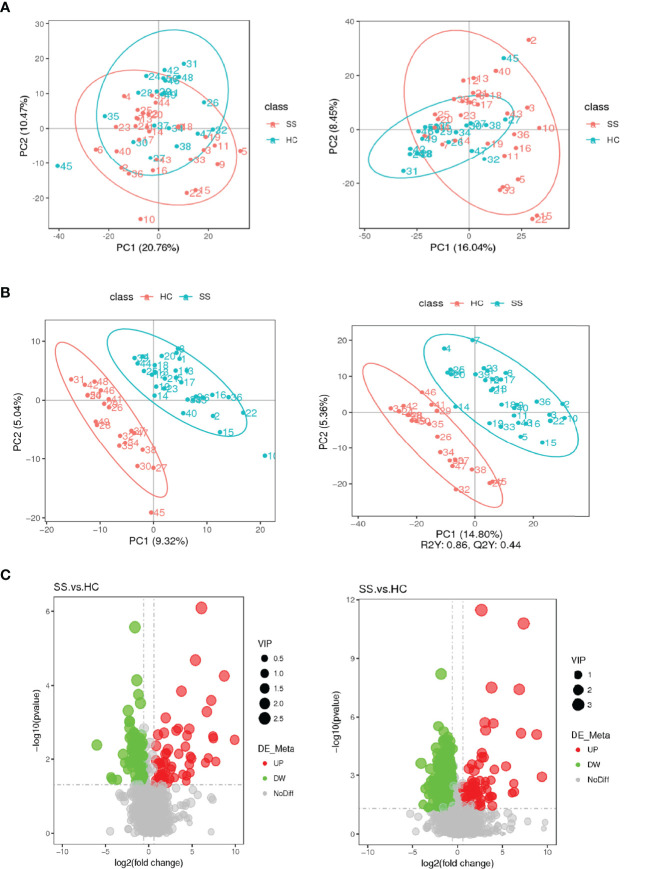
Score plots of the PCA and PLS-DA models and volcanic map of differential metabolites. **(A)** Score plot of the PCA model in the ESI- and ESI+ ion modes. **(B)** Score plot of the PLS-DA model in the ESI- and ESI+ ion modes. **(C)** Volcanic map of differential metabolites. The horizontal coordinate represents the fold change (log2FoldChange) of metabolites in different groups, and the vertical coordinate represents the significance level of the difference (-log10P value). Each point in the volcano map represents a metabolite, in which the metabolites with significantly up-regulated expression are represented by red dots, and those with significantly down-regulated expression are represented by green dots. The dot size VIP values are represented by dot size.

The score plot of the PCA model was used to analyze the first three principal components of the two groups, which indicated that the principal components were separated effectively. In addition, PLS-DA, which exhibits a better discrimination ability than that of PCA (22), was also conducted to analyse the metabolic profiles based on class information. The PLS-DA model showed significant clustering, indicating there was obvious separation between the two groups. (**Figure 4**)

**Figure 5:**
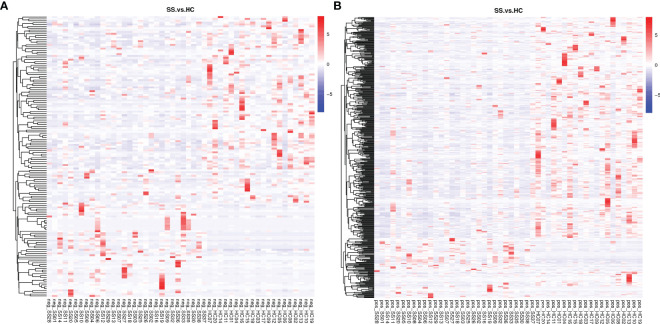
Heat map of the differential metabolites in pSS patients and HCs. **(A)** ESI-, **(B)** ESI+. The colors from blue to red indicate the relative contents of the metabolites in the two groups.

A total of 2051 metabolites in ESI+ mode and 923 metabolites in ESI- mode were identified in pSS patients and HCs (n=50) (**Additional File 1: Supplementary Table S1**), of which 459 metabolites in ESI+ mode and 152 metabolites in ESI- mode were subjected to statistical analysis. Global overview of differential metabolism features was shown in the heat map. (**Figure 5**) Based on variable importance in the projection (VIP) values >1 in the loading plot, FC≧2 or FC≦0.5, and *P*<0.05 and with KEGG annotations, there were 33 differentially accumulated metabolites were identified, of which 13 metabolites were enriched and 20 metabolites were depleted (**Additional File 1: Supplementary Table S2**). These metabolites were thus selected as a reference for further analyses. In addition, we found through KEGG enrichment analysis that these metabolites were mainly involved in amino acid metabolism, including histidine, phenylalanine, tyrosine and tryptophan metabolism, and in lipid metabolism, including arachidonic acid and steroid biosynthesis.

### 3.4 Cross-Correlation Analysis Between the Microbiota and Metabolites

**Figure 6:**
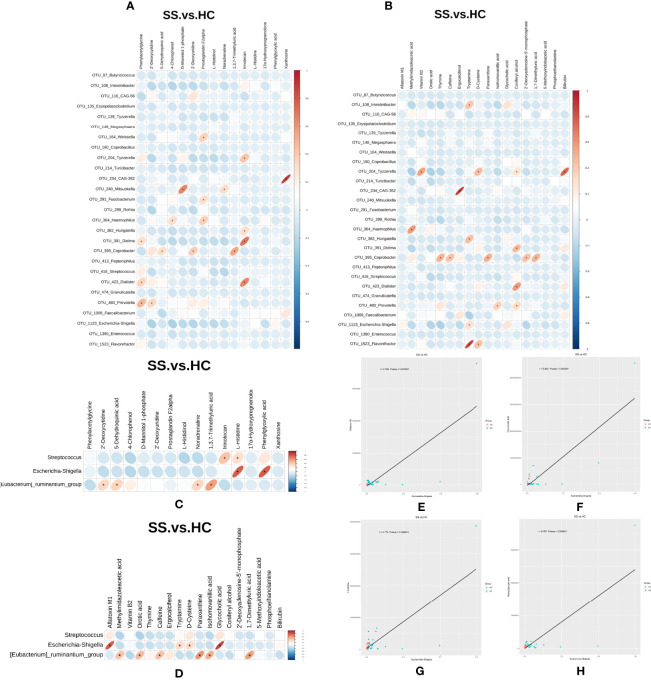
Cross-correlation analysis between microbiota and metabolites. **(A, B)** Heatmap of the correlation between the top 28 OTUs with statistical difference annotated at the genus level and metabolites in the ESI- and ESI+ ion modes. **(C, D)** Heatmap of the correlation between the three microbiotas in genus level and metabolites in the ESI- and ESI+ ion modes. The horizontal coordinate represents the differentially abundant bacteria at the level of the 16S gene, and the vertical coordinate represents the differentially abundant metabolites. Red indicates a positive correlation, blue indicates a negative correlation, and an asterisk (*) indicates statistical significance, that is, *P* < 0.05. **(E–H)** Scatter plot of the correlation between *Escherichia-Shigella* and four metabolites (aflatoxin M1, glycocholic acid, L-histidine and phenylglyoxylic acid).

In order to explore the functional relationship of the altered gut microbiota and differentially accumulated fecal metabolites, we performed correlation analysis based on Pearson’s correlation coefficients. The top 28 OTUs with statistical difference annotated at the genus level, and the above 33 differentially accumulated metabolites were included for analysis. It showed the metabolites were correlated with the microbiota of pSS patients. The heatmap of the correlation is shown in **Figures 6A, B**. Besides, the results of the above microbiota study showed that *Escherichia-Shigella*, *Eubacterium-ruminantium*, and *Streptococcus* in genus level were related to SS. We also made the cross-correlation analysis between the three microbiotas with the differentially accumulated metabolites (**Figures 6C, D**).

The Pearson correlation coefficient (r) and P value were used to analyze the relevance of the relative abundance of metabolites and microorganisms. | r | ≧0.6 and *P* ≤0.05 were considered to indicate statistical significance. We found that the abundance of *Escherichia-Shigella* was correlated with high levels of four metabolites (aflatoxin M1, glycocholic acid, L-histidine and phenylglyoxylic acid) (**Figures 6E–H**).

## 4 Discussion

Our research focused on the microbiological and metabolic characteristics of Chinese pSS patients. We found that (1) pSS patients had increased levels of proinflammatory gut microbes and decreased levels of anti-inflammatory gut microbes; (2) pSS patients had unique metabolic characteristics; and (3) there were certain correlations between the microbiota and metabolism in pSS patients.

In terms of the gut microbiota, we found some differences in gut microbiota diversity, richness and evenness in pSS patients compared with healthy people. There was no significant agreement between the alpha- and beta-diversity analyses. However, the bacterial abundances differed at the phylum and genus levels.

At the phylum level, we found that *Firmicutes* was the dominant member in the gut microbiota, followed by *Bacteroidetes. Firmicutes* and *Bacteroidetes* are the two major bacteria in the gut microbiota and play important roles in the maintenance of intestinal homeostasis. These two bacterial phyla have been linked to a variety of diseases (23, 24). In line with the study by Moon et al. (10), we also found a decreased *Firmicutes*-*Bacteroidetes* ratio in pSS patients compared with controls. A shift in the *Firmicutes*-*Bacteroidetes* ratio is known to be the first indication of gut dysbiosis. A reduced *Firmicutes*-*Bacteroidetes* ratio has also been observed other chronic autoimmune diseases, such as SLE and systemic sclerosis (10). In addition, we also found that pSS patients exhibited enrichment of *Proteobacteria* and *Actinobacteriota*, which was consistent with the results of Mendez et al.’s (1) research.

At the genus level, our study indicated that pSS patients had significantly increased abundances of *Escherichia-Shigella*, *Veillonella* and *Bacteroides*. As reported, *Escherichia-Shigella* was found to be enriched in pSS model mice generated by dehydration and antibiotics (25). Research has shown that *Escherichia-Shigella* is associated with proinflammatory states (26). Cattaneo et al. (27) found that the abundance of *Escherichia-Shigella* was positively correlated with levels of proinflammatory molecules, including IL-6 and NLRP3. De La Fuente et al. (28) reported that *Escherichia* can induce the production of proinflammatory cytokines through an NLRP-3-dependent mechanism. *Veillonella*, a genus of potential pathobionts, was reported to be closely associated with primary sclerosing cholangitis and autoimmune hepatitis (29, 30). The abundance of *Veillonella* was reported to be synergistically or interactively associated with elevated IL-1β, IL-8, and IgA levels in IBD patients (31).

In our study, pSS patients revealed a reduced abundance of the genus *Faecalibacterium* compared to the control group. At the family level, we found that *Ruminococcaceae* and *Lachnospiraceae* were depleted in pSS patients. *Faecalibacterium*, a member of Clostridium IV (*Firmicutes*), is an abundant bacterial genus in the gut microbiome of healthy people and can produce SCFAs (including butyrate). Our research showed that the abundance of *Faecalibacterium* was significantly decreased in pSS patients compared with controls, which is consistent with de Paiva et al.’s (25) research. They found a decrease in *Faecalibacterium* abundance in both pSS patients and pSS model mice. Research found that in IBD patients, disturbances in an association network containing *Ruminococcaceae* and *Lachnospiraceae* frequently characterize relapsing disease and poor responses to treatment with anti-TNF therapeutic antibodies (32). In addition, the intestinal abundance of *Lachnospiraceae* was reported to be decreased in a rat model with irritable bowel syndrome (33) and chronic pancreatitis (34). *Ruminococcaceae* and *Lachnospiraceae* are taxa that can induce the production of SCFAs. SCFAs, especially butyrate, play an important role in immunity status, enhancing the intestinal mucosal immune barrier by promoting the activity of Treg cells and IL-10 production. In addition, they also inhibit the release of proinflammatory cytokines such as IL-2, IL-8 and TNF, thus exerting an effective anti-inflammatory effect (35). The decrease in the levels of the above flora in pSS patients may lead to a decrease in butyrate concentration in the body; thus, the balance between proinflammatory factors and anti-inflammatory factors is destroyed, and the immune balance is affected, which results in inflammation.

Based on random forest analysis, *Escherichia-Shigella*, *Eubacterium-ruminantium* and *Streptococcus* were confirmed to be significantly predictive markers for pSS. The abundance of *Escherichia-Shigella* and *Streptococcus* was elevated in pSS patients, while that of *Eubacterium-ruminantium* was decreased in pSS patients. One study on the gut microbial profiles of AS patients also found a lower abundance of *Eubacterium-ruminantium* in AS patients (36). *Eubacterium-ruminantium* from *Firmicutes* is usually present in the gut microbiota of healthy hosts, maintaining the health of the host and acting as a probiotic by regulating the balance of the intestinal environment (37). Studies have found that the abundance of *Streptococcus* is increased in inflammatory diseases (36, 38, 39). *Streptococcus* is an opportunistic pathogen and may trigger the release of proinflammatory factors and induce chronic inflammation.

Thus, we concluded that pSS patients have increased abundances of proinflammatory gut microbes, including *Escherichia-Shigella*, *Veillonella*, Bacteroides and *Streptococcus*, and decreased abundances of anti-inflammatory gut microbes, including *Faecalibacterium*, Ruminococcaceae, *Lachnospiraceae* and *Eubacterium-ruminantium.*

With regard to the metabolome, many studies have revealed that altered metabolite profiles are associated with various diseases, such as RA (16), SADs (40), and depression (41), but few studies have focused on SS. In our study, the metabolomic analysis indicated that pSS patients had unique metabolic characteristics compared to healthy people. Compared with the metabolomics study performed by Li et al. (16), the common differential metabolite bilirubin was found. In that study bilirubin in pSS patients was slightly lower than HC, but in our study, bilirubin in pSS patients was slightly higher than HC, which may be related to sample differences. Feces were used in our study, while serum was studied in that study. The content of the metabolites in serum can be further detected for further investigation.

The PLS-DA analysis showed that the two groups were well separated. A total of 33 differentially accumulated metabolites were identified. Through KEGG enrichment analysis, we found that the metabolites were mainly involved in amino acid metabolism, including histidine, phenylalanine, tyrosine and tryptophan metabolism, and lipid metabolism, including arachidonic acid and steroid biosynthesis.

It has been confirmed that changes in the serum levels of amino acids could reflect the presence of osteoarthritis in the knee (42). One study showed that the levels of several amino acids were slightly changed in RA patients; they found that the levels of L-leucine, L-phenylalanine, glutamic acid and L-proline were significantly increased, while those of tryptophan and argininosuccinic acid were decreased, in RA patients (16).

In our study, we found that the levels of L-histidine, phenylglyoxylic acid, and homovanillic acid from tyrosine metabolism were elevated in pSS patients, while the level of 5-methoxyindoleacetic acid from tryptophan metabolism was decreased. It has been reported that L-histidine could participate in the regulation of the blood coagulation pathway by affecting platelet function. Studies have shown that patients with pSS could develop coagulation disorders (43). Homovanillic acid (HVA) is a terminal metabolite of catecholamines. Catecholamines can drive humoral immunity by stimulating macrophage IL-10 production. The action of these catecholamines is mediated primarily by beta (2)-AR activation (44). Research has shown that the tryptophan level is decreased in the plasma of patients with AS compared with healthy people. The release of tryptophan from its binding serum protein is a sign of satisfactory curing of AS. A lower level of tryptophan in AS patients might be an indicator of disease progression (45), which could also explain the situation in pSS patients.

Changes in lipid profiles have been reported to be associated with many diseases, such as RA (46) and gastric cancer (22). Arachidonic acid is the fatty acid precursor of prostaglandins and other eicosanoids linked to inflammation. The presence of a high ratio of arachidonic acid may be responsible for the increased incidence of arthritis and other chronic inflammatory diseases (47). One study aimed to investigate the effects of eicosapentaenoic (EPA) and docosahexaenoic acids (DHA) on acute inflammation by feeding rats an EPA-rich diet and DHA-rich diet, and the carrageenan-induced swelling of footpads was measured at the end. They found that the mean ratio of arachidonic acid to the sum of highly unsaturated fatty acids was correlated with the mean degree of swelling among all dietary groups (48).

Recently, metabolomic changes in fecal samples have been reported to be associated with the gut microbiota in the development of diseases, such as diabetes (49), Crohn’s disease (50), and systemic sclerosis (51). In our study, we found certaincorrelations between the gut microbiota and fecal metabolites through Pearson’s correlation analysis. Although we could not identify a definite causal relationship through this analysis, we found some correlations between the two. In particular, we found that the abundance of *Escherichia-Shigella* was correlated with high levels of four metabolites (aflatoxin M1, glycocholic acid, L-histidine and phenylglyoxylic acid). These results indicate that amino acid metabolism is overactive during the development of pSS, which may be closely related to gut microbiota function.

Some limitations of our research should be acknowledged. First, the total sample size was small. SS is a heterogeneous disease with different phenotypes, but the gut microbiota and metabolomic characteristics of different phenotypes were not reflected. Second, the influence of environmental, dietary, geographic and other factors on the microbiota and metabolomic results should also be taken into account. We cannot ensure that our discoveries will apply to genetically diverse populations with different lifestyles and diets. Third, no comparison was made between Sjogren’s Syndrome and other rheumatic disease. Finally, the observed results were not validated in animal models to better define the functional role of the identified microbiota and metabolites.

## 5 Conclusions

The gut microbiota and fecal metabolic phenotype in pSS patients were measured through 16S rRNA gene sequencing and LC–MS methods. Our research concluded that pSS patients had not only a significantly different gut microbiota but also significantly different fecal metabolites. In addition, correlation analysis indicated that the changes in some gut microbes were correlated with changes in metabolites. In conclusion, pSS not only disturbs the gut microbiota at the abundance level but also alters the host’s metabolic homeostasis. In general, regulated gut microbiota-related metabolites may serve as a new entry point for mechanistic research on pSS and as a tool for early prediction, diagnosis and treatment tools.

## Data Availability Statement

The datasets presented in this study can be found in online repositories. The names of the repository/repositories and accession number(s) can be found below: Sequencing data can be found at NCBI SRA BioProject, accession no: PRJNA814076; Mass spectrometry data can be found at ProteomeXchange Consortium, accession no: PXD032289.

## Ethics Statement

The studies involving human participants were reviewed and approved by the Ethics Committee of Mianyang Central Hospital, School of Medicine, University of Electronic Science and Technology of China. The patients/participants provided their written informed consent to participate in this study.

## Author Contributions

LY, JZ, and JY designed the research studies. ZX, YZ, and YN provided samples. LY, ZX, YZ, and YN performed the research and analyzed the data. LY, ZX, and JZ wrote the paper. All authors read and approved the final manuscript.

## Funding

This work was supported by the Key Research and Development Project of Science and Technology Department of Sichuan Province (2017SZ0148), the Incubation Project of Mianyang Central Hospital (2020FH10) and the Medical Science and Technology project of Sichuan Provincial Health Commission (21PJ180).

## Conflict of Interest

The authors declare that the research was conducted in the absence of any commercial or financial relationships that could be construed as a potential conflict of interest.

## Publisher’s Note

All claims expressed in this article are solely those of the authors and do not necessarily represent those of their affiliated organizations, or those of the publisher, the editors and the reviewers. Any product that may be evaluated in this article, or claim that may be made by its manufacturer, is not guaranteed or endorsed by the publisher.

## Abbreviations

LC–MS, liquid chromatography–mass spectrometry; UHPLC–MS, ultrahigh-performance liquid chromatography–mass spectrometry; pSS, primary Sjogren’s syndrome; HCs, healthy controls; HCV, hepatitis C virus; HBV, hepatitis B virus; DC, dendritic cells; SCFAs, short-chain fatty acids; ESSDAI, EULAR Sjogren’s syndrome disease activity index; ClinESSDAI, Clinical EULAR Sjogren’s syndrome disease activity index; SADs, systemic autoimmune diseases; AS, ankylosing spondylitis.
