# Supplementary material for: Comprehensive Analysis of the Relationships Between the Gut Microbiota and Fecal Metabolome in Individuals With Primary Sjogren’s Syndrome by 16S rRNA Sequencing and LC–MS-Based Metabolomics
Source: Front Immunol. 2022 May 11;13:874021. doi: 10.3389/fimmu.2022.874021 (PMC9130595; doi:10.3389/fimmu.2022.874021)
Supplement: Supplementary file 1 [file DataSheet_1.docx]

Supplementary Material

## Supplementary Figures

Gut microbiota and metabolome in pSS patients

Healthy controls (n=20)

2020.11 to 2021.02

pSS patients (n=30)

2020.05 to 2021.02

Exclusion criteria:

1. age ＜18 years
2. concurrent inflammatory bowel disease (IBD)/severe diarrhea.
3. antibiotic treatment in the last 3 months.

Results of metabolic analysis

Results of gut microbiota analysis

Correlation analysis between microbiota and metabolites

Identification of the difference between pSS patients and HCs

**Supplementary Figure 1**. Flow diagram of this study.
